# Accelerating Fourth-Generation Machine Learning Potentials Using Quasi-Linear Scaling Particle Mesh Charge Equilibration

**DOI:** 10.1021/acs.jctc.4c00334

**Published:** 2024-08-16

**Authors:** Moritz Gubler, Jonas A. Finkler, Moritz R. Schäfer, Jörg Behler, Stefan Goedecker

**Affiliations:** † Department of Physics, University of Basel, Klingelbergstrasse 82, CH-4056 Basel, Switzerland; ‡ Lehrstuhl für Theoretische Chemie II, Ruhr-Universität Bochum, 44780 Bochum, Germany; ¶ Research Center Chemical Sciences and Sustainability, Research Alliance Ruhr, 44780 Bochum, Germany

## Abstract

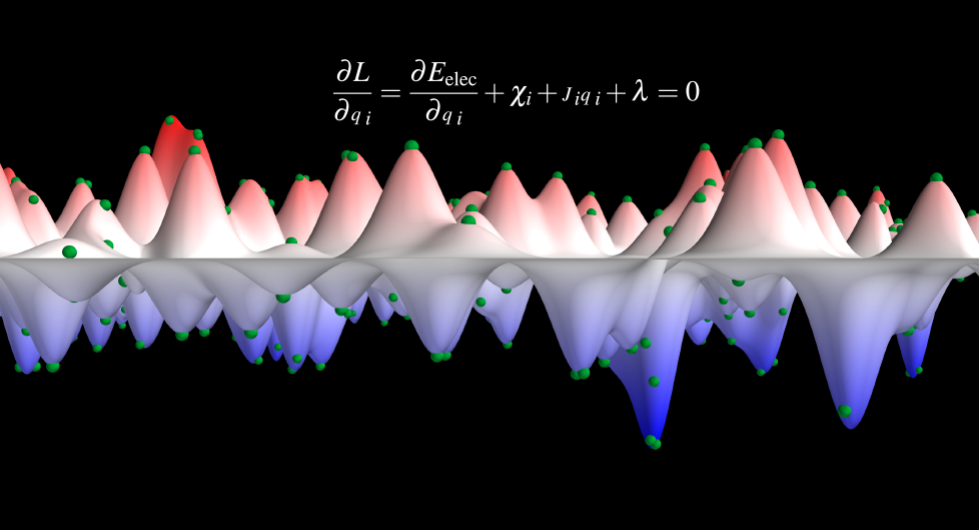

Machine learning potentials (MLPs) have revolutionized the field of atomistic simulations by describing atomic interactions with the accuracy of electronic structure methods at a small fraction of the cost. Most current MLPs construct the energy of a system as a sum of atomic energies, which depend on information about the atomic environments provided in the form of predefined or learnable feature vectors. If, in addition, nonlocal phenomena like long-range charge transfer are important, fourth-generation MLPs need to be used, which include a charge equilibration (Qeq) step to take the global structure of the system into account. This Qeq can significantly increase the computational cost and thus can become a computational bottleneck for large systems. In this Article, we present a highly efficient formulation of Qeq that does not require the explicit computation of the Coulomb matrix elements, resulting in a quasi-linear scaling method. Moreover, our approach also allows for the efficient calculation of energy derivatives, which explicitly consider the global structure-dependence of the atomic charges as obtained from Qeq. Due to its generality, the method is not restricted to MLPs and can also be applied within a variety of other force fields.

## 1 Introduction

Obtaining accurate potential energy surfaces (PESs) at a reasonable computational expense is one of the greatest challenges in computational chemistry, physics, and materials science. Since even the most efficient electronic structure methods like density functional theory (DFT) are usually too demanding for large-scale simulations, the conventional approach taken in computer simulations of complex systems involves the use of heuristically derived force fields and empirical potentials. These are often able to capture the main features of the atomic interactions but are quantitatively less accurate than first-principles methods based on the direct solution of the quantum mechanical equations.

In recent years, the rapid development of data-driven machine learning potentials (MLPs), which offer accurate PESs with only a fraction of the computational costs of the underlying electronic structure calculations used in the training process, has paved the way to solve this dilemma.^1−8^ A key step in the development of MLPs for large condensed systems has been the construction of the total energy as a sum of atomic energies, which only depend on the atomic environments up to a cutoff radius.^9^ Many flavors of such local, second-generation MLPs^10^ have been proposed and successfully applied to a variety of systems to date.^9,11−16^ By construction, they show a favorable, essentially linear scaling with system size. In addition, third-generation MLPs include long-range electrostatic interactions based on environment-dependent charges represented by machine learning.^17−21^ In spite of including these long-range electrostatic interactions without truncation, such third-generation MLPs are still “local” in the sense that they are unable to take nonlocal phenomena like long-range charge transfer beyond the local atomic environments into account.^22^

The need to consider long-range charge transfer, which is present in a variety of systems, in atomistic potentials has attracted a lot of attention for several years. For instance, charge equilibration (Qeq), which was initially developed by Rappe and Goddard III^23^ and later refined by Nakano,^24^ is a well-established method to approximate complicated electrostatics and charge transfer effects. As shown by Grimme et al.,^25^ charge equilibration techniques can also be used in the context of dispersion corrections. Warren et al.^26^ demonstrated that the polarizability of long molecules is severely overestimated in the original Qeq approach^23^ and proposed the addition of charge constraints for subsystems to overcome this problem. An overview and comparison of popular charge equilibration methods has been provided by Ongari et al.,^27^ and nowadays modern variants of Qeq are routinely used in advanced force fields such as ReaxFF^28^ or COMB^29^ and are available in widely distributed simulation software packages like LAMMPS.^30^

The first use of charge equilibration in the framework of MLPs was the charge equilibration neural network technique (CENT) introduced in 2015 by Ghasemi et al.,^31^ and this first fourth-generation MLP has been further improved in the following years.^32,33^ In CENT, the atomic electronegativities of the charge-equilibration approach are expressed as environment-dependent atomic properties learned by atomic neural networks, with the goal of reproducing the correct total energy of the system. Due to the underlying total energy expression, the CENT approach is best suited for a description of systems with primarily ionic bonding. A fourth-generation high-dimensional neural network potential (4G-HDNNP) that is more generally applicable to all types of systems has been proposed by Ko et al.^34^ by combining the advantages of CENT and second-generation HDNNPs. Here, the neural networks providing the atomic electronegativities are trained to reproduce reference atomic partial charges, and the resulting electrostatic energy is combined with modified atomic neural networks contributing atomic energies representing local bonding that explicitly takes charge transfer into account.

Incorporating long-range charge transfer and the resulting electrostatics into machine learning models has become increasingly popular over the last years, and many other approaches have been proposed.^35−41^ Still, so far, the application of fourth-generation MLPs has been restricted to small- and medium-sized systems, primarily due to the high computational cost of solving a set of linear equations, which is needed in the original charge equilibration method. In order to improve the scaling of charge equilibration, Nakano^24^ proposed a multilevel conjugate gradient approach that solves the set of equations iteratively. Because this approach requires the calculation of the Coulomb matrix, which is dense, the scaling is at least quadratic with respect to the number of atoms. The performance of iterative charge equilibration schemes can be enhanced by algorithms that do not require explicit knowledge of the matrix elements, as shown by Rostami et al.^42^ The efficiency of such an iterative scheme can be further improved if it is combined with the conjugate gradient method that allows a reduction of the number of iterations compared to a steepest descent approach.

Apart from the determination of the atomic charges, another important aspect that has to be considered in the development of more efficient methods is that fourth-generation MLPs often make use of the charges obtained from the charge equilibration step as input for calculating energy terms beyond simple electrostatics, which consequently also need to be taken into account in the determination of atomic forces and the stress tensor.^34^ This requires additional steps that also need to be implemented in a computationally efficient way.

In this work, we propose a formulation of the charge equilibration method, which combines a rapidly converging conjugate gradient with matrix times vector multiplications that do not require explicit knowledge of the Coulomb matrix elements. This results in quasi-linear scaling with respect to the number of atoms in the system. Moreover, with our ansatz, it is possible to calculate derivatives of energy terms that use charges obtained from charge equilibration as input in the quasi-linear time. Our method is therefore ideally suited for a combination with 4G-HDNNPs^34^ and numerous other types of MLPs that depend on the atomic charges.

After a concise summary of the theoretical background in section 2, we derive the equations for the efficient calculation of the electrostatic energy, forces, and stress for periodic systems using our particle mesh charge equilibration method in section 3. This approach is also expected to be very useful in other contexts requiring the solution of Poisson’s equation in case the charge density has the same form of a smooth superposition of generic atom-centered spherically symmetric charge densities. After a discussion of our results for a reference implementation in section 4, we conclude our main findings in section 5.

## 2 Theoretical Background

### 2.1 Charge Equilibration

In the charge equilibration formalism, the energy of the system *E*_Qeq_ is defined as the sum of the electrostatic energy *E*_elec_ and a Taylor expansion of atomic energies that depend on atomic charges **Q** = {*q*_*i*_}
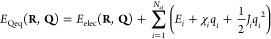
1with **R** = {**r**_*i*_} being the atomic coordinates and {*E*_*i*_} being the element-dependent atomic reference energy offsets. These energy offsets *E*_*i*_ cause a shift in *E*_Qeq_ but do not change the charges that are obtained by using the charge equilibration method. In eq 1 the Taylor expansion is truncated after the second-order terms, and the expansion coefficients {χ_*i*_} and {*J*_*i*_} are called electronegativity and hardness, respectively. Moreover, the electrostatic energy is computed as *E*_elec_ = 1/2 ∫ ρ(**r**)*V*(**r**)d**r** and the charge density
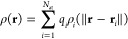
2is a superposition of atomic charge densities *q*_*i*_ρ_*i*_ that are spherically symmetric around the position of atom *i*. The charge distributions ρ_*i*_ are normalized to 1, i.e., ∫ ρ_*i*_ d**r** = 1, and scaled by the respective atomic charge *q*_*i*_. *V*(**r**) is the electrostatic potential of charge density ρ. The atomic charge densities are usually chosen to be either point charges or Gaussian charge distributions with an element-specific width σ_*i*_.

Charge equilibration is defined as the minimization of *E*_Qeq_ with respect to atomic charges **Q** under the constraint of keeping the total charge *Q*_tot_ of the system constant. Using the method of Lagrange multipliers, the function
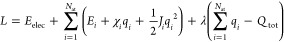
3has to be stationary. Differentiating eq 3 with respect to *q*_*i*_ and λ yields the set of equations
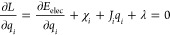
4
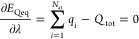
5Inserting the definition of the charge density into the electrostatic energy yields
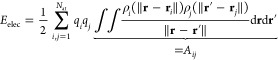
6The matrix **A** is symmetric and positive definite because *E*_elec_ = 1/2**Q**^*T*^**AQ** ≥ 0 and *E*_elec_ = 0 ⇔ **Q** = 0. The first inequality holds because the electrostatic interaction energy of a smooth charge density is always positive. Because of the spherical symmetry of the Gaussian density ρ_*i*_ around atom *i*, *A*_*ij*_ is a function of the distance between **r**_*i*_ and **r**_*j*_. Using the definitions from eqs 4 and 6 and , the derivative of the energy with respect to the atomic charges can be simplified to
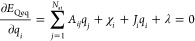
7Equations 4 and 5 can be written in matrix notation as follows:
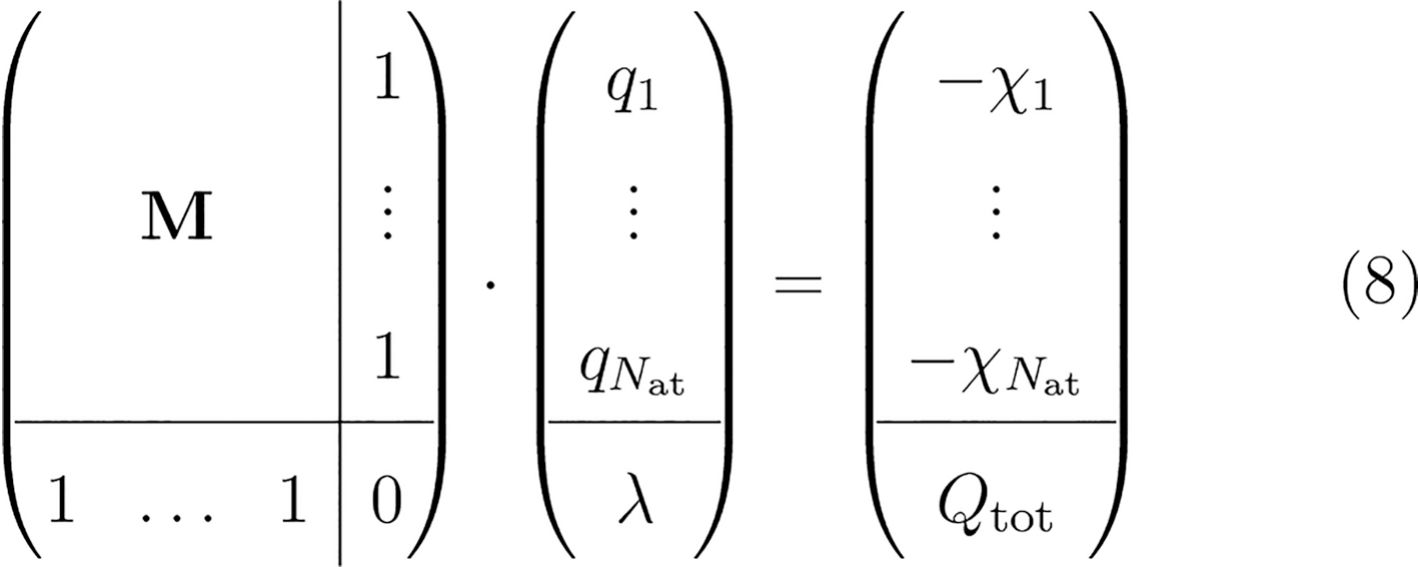
8where
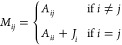
9This set of linear equations can be solved either directly with cubic scaling or using the iterative multilevel conjugate gradient approach.^24,42^ Since matrix **A** has to be calculated, the best scaling using the latter approach is  because the Coulomb matrix is dense and has *N*_at_^2^ elements.

### 2.2 Calculation of Total Derivatives

Since the charge equilibration energy depends on the atomic positions, both explicitly and implicitly, via the dependence of the charges on the atomic positions, the gradient is given by the expression
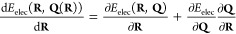
10Since in charge equilibration the energy *E*_Qeq_ is minimized with respect to the *q*_*i*_, we have  and hence the total derivative simplifies to
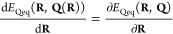
11The situation is more complicated if the charges are not determined by a minimization of the total energy. For example, in 4G-HDNNPs^34^ the charges obtained by the charge equilibration are used as parameters to calculate the short-range and electrostatic energies. In that case, the gradient of the total energy of the system is given by
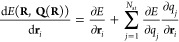
12and the derivatives  are required. In total, there are 3*N*_at_^2^ derivatives of atomic charges with respect to atomic positions, which would result in at least quadratic scaling when the derivatives are calculated. However, the total derivative  can be evaluated without calculating  explicitly. A similar approach has been used by Poier et al.^43^ in the context of polarizable force fields. Ko et al.^34^ derived an efficient way to calculate the forces in the 4G-HDNNP method. The resulting formulas for the forces and the strain derivatives  needed for calculating the stress are
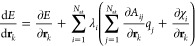
13
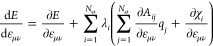
14where **λ** can be obtained by solving
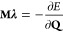
15under the constraint that the sum of the components of **λ** is 0.

In the derivation of the total derivatives, it is assumed that the electronegativities χ_*i*_ depend on the atomic positions, whereas the hardnesses *J*_*i*_ are only element-specific quantities. While the derivation could also be extended to consider position-dependent hardnesses, we assume that the hardness is an element-specific parameter throughout this Article.

The cost of evaluating eqs 13 and 14 directly still scales at least quadratically. In section 3.4, it is discussed how these sums can be evaluated more efficiently.

## 3 Particle Mesh Charge Equilibration

### 3.1 Solving the System of Equations

We start by noting that, as shown in section 1 in the Supporting Information,  can be written as
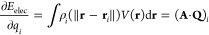
16With this relation, the matrix-vector product **A**·**Q** can be calculated for an arbitrary **Q** without any explicit knowledge about the elements of matrix **A**. Being able to calculate matrix-vector products for arbitrary vectors is sufficient to solve a system of equations iteratively.

As discussed in section 2.1, the Coulomb matrix is positive definite. The manifold of the charge equilibration constraint ∑_*i*_*q*_*i*_ = *Q*_tot_ is a hyperplane, meaning that an arbitrary large step along a constrained gradient will still fulfill the charge conservation constraint. Therefore, the standard conjugate gradient method can be used to solve the set of linear equations.
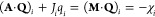
17where
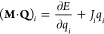
18

The constrained gradient 
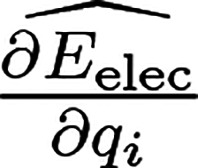
 of eq 16 can be obtained by projecting the gradient onto the constraint
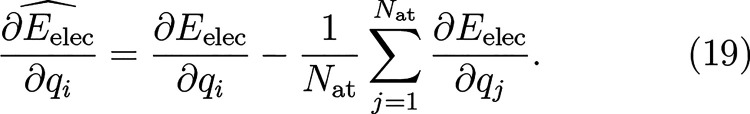
19

### 3.2 Plane Wave Methods

In order to minimize *E*_Qeq_ quickly, it is necessary to evaluate eq 16 efficiently. In the case of periodic boundary conditions, this can be done by solving Poisson’s equation in Fourier space using plane waves. Let ρ̃(**G**) and *Ṽ*(**G**) be the Fourier transforms of ρ and *V*, respectively, and **G** is a Fourier space vector. Because of the Plancherel theorem, *E*_elec_ can be calculated in Fourier space as
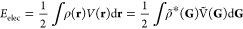
20where the superscript * of ρ represents complex conjugation. This is particularly useful because Poisson’s equation can be solved analytically in Fourier space with the solution . The electrostatic energy can be calculated by Fourier transforming ρ and then solving the Fourier space integral in eq 20. Then, the electrostatic potential *V*(**r**) in real space can be obtained efficiently by back-transforming the Fourier coefficients .

In case of periodic boundary conditions, the integral in eq 20 transforms into the following series:
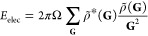
21where Ω is the unit cell volume. Also, Fourier transforms of any periodic function can be obtained numerically using the Fast Fourier transform (FFT), which can be calculated with a  scaling where *N* is the number of gridpoints.

The electrostatic potential *V*(**r**) can be obtained by using a forward and backward Fourier transform. The atomic charge densities ρ_*i*_, which are present in eq 16, typically decay exponentially, which makes it possible to obtain  for all atoms in quasi-linear time because it is sufficient to integrate only over a small volume around each atom *i* to obtain . Therefore, the matrix vector product  can be evaluated for any **Q** in quasi-linear time.

### 3.3 Derivatives with Plane Wave Methods

Most materials science simulations require the calculation of the forces acting on the nuclei and the stress tensor acting on the periodic lattice. Using the definition of ρ from eq 2, the electrostatic force can be obtained by evaluating the real space integral
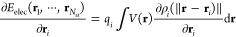
22which is derived in section 2 in the Supporting Information.

Regarding the electrostatic stress, two different definitions of stress are commonly in use: the microscopic stress and the macroscopic stress. The microscopic stress tensor is a tensor field, and an example is the Maxwell stress  for systems without periodic boundary conditions. For periodic systems, the microscopic stress tensor is rarely used, since in bulk materials it is often sufficient to consider the macroscopic stress tensor, which corresponds to the average microscopic stress per unit volume. The symmetric strain tensor ε_*μν*_ describes an infinitesimal deformation of a crystal *r*_μ_^′^ = (δ_*μν*_ + ε_*μν*_)*r*_ν_, where δ stands for the Kronecker δ and the Einstein summation convention is used. The macroscopic stress **σ** is the strain derivative of the total energy per unit volume^44,45^ with
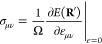
23**R**′ contains the atomic positions **R** that are deformed with the strain tensor ε_*μν*_. Because of the variational character of electronic structure calculations, the strain derivative of the charge density is zero, which reduces eq 23 to the average Maxwell stress that can easily be calculated when the total potential *V*(**r**) is known. The strain derivative of the charge density is not zero in our case, and the total stress has to be evaluated in Fourier space where  is given by
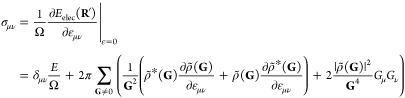
24A derivation of eq 24 can be found in section 3 in the Supporting Information. The Fourier transform of the strain derivative  can be obtained by transforming the strain derivatives  of the charge density into Fourier space, which requires six additional Fourier transforms as  is symmetric. The periodic generalization of eq 2 for the lattice matrix **h** containing the three lattice vectors **h**_1_, **h**_2_, and **h**_3_ is given by
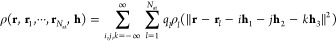
25The calculation of the strain derivative of eq 25 is discussed in section 4 in the Supporting Information. It has the following form:
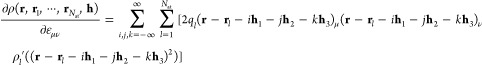
26The sums over *i*, *j*, and *k* in eqs 25 and 26 are over all periodic images of the simulation cell.

### 3.4 Particle Mesh Total Derivatives

Using the definitions in eq 27, the corresponding potentials *V*^**Q**^ and *V*^**λ**^, and the electrostatic energies in eq 28,
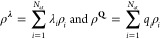
27
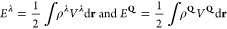
28the double sums in eqs 13 and 14 can be expressed in terms of the newly introduced variables as
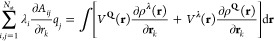
29and
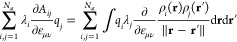
30Equation 29 can be solved in real space, and eq 30 can be solved in Fourier space, in which the integral has the form
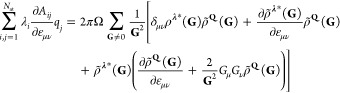
31which can all be evaluated with quasi-linear scaling. A derivation of eqs 29 and 31 is presented in section 5 in the Supporting Information.

The coefficients λ_*i*_ can be obtained by solving , where *E* is an arbitrary energy that depends on charges obtained by minimizing *E*_Qeq_. Once again, the matrix-vector product  can be evaluated without knowledge of the elements of **A**. Therefore, it can also be solved iteratively using the conjugate gradient method.

## 4 Results and Discussion

Overall, our iterative particle mesh charge equilibration method can be summarized as follows:

1. *Charge equilibration:* Solve **M**·**Q** = −**χ** under the constant charge constraint using the conjugate gradient method. .
2. *Calculate***λ***charges*: Solve  under the constraint that ∑_*i*_λ_*i*_ = 0 using the conjugate gradient method.
3. *Calculate forces and stress:* Use eqs 13, 14, 29, and 31 to calculate the forces and the stress tensor.

**Figure 1:**
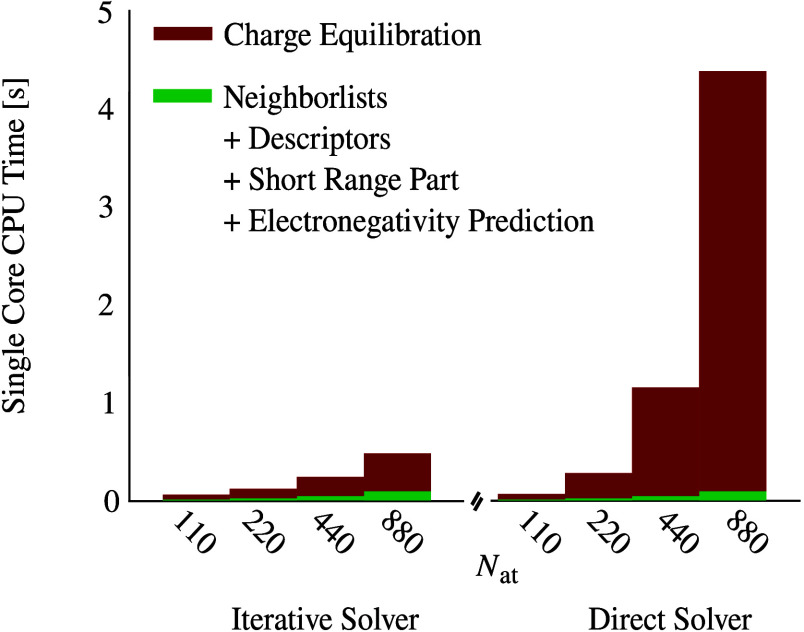
Benchmark results illustrating the performance of the iterative particle mesh electrostatic method and the conventional direct method. The figure displays the average single core CPU times required for predicting the energies of 50 randomly selected periodic structures from a data set containing Au_2_ clusters on undoped and doped MgO(001) surfaces described by Ko et al.^34^ Each structure comprises 110 atoms, along with their respective supercells containing 220, 440, and 880 atoms. The benchmark includes both the direct method employed in their study on the right and the newly introduced iterative particle mesh method on the left. The simulations were conducted using the RuNNer code^46,47^ on a single-core CPU on a Intel Xeon 6430 processor with 32 cores operating at 2.10 GHz and a 270W TDP. The system is equipped with 512 GB DDR5-4800 ECC REG RAM (16·32 GB).

In a first step, we have compared the computational efficiency of the particle mesh charge equilibration method with the conventional charge equilibration approach, i.e., the direct solution of a set of linear equations. For this purpose, the presented iterative particle mesh electrostatic method has been incorporated into our MLP software RuNNer,^46,47^ yielding a significant enhancement for molecular simulations employing 4G-HDNNPs. Results, comprehensively presented in Figure 1, show large performance gains for the new iterative electrostatic approach compared to the conventional direct method. Examination of the computation time distribution reveals the significant contribution of the electrostatic component, highlighting its dominance in the overall computational costs.

**Figure 2:**
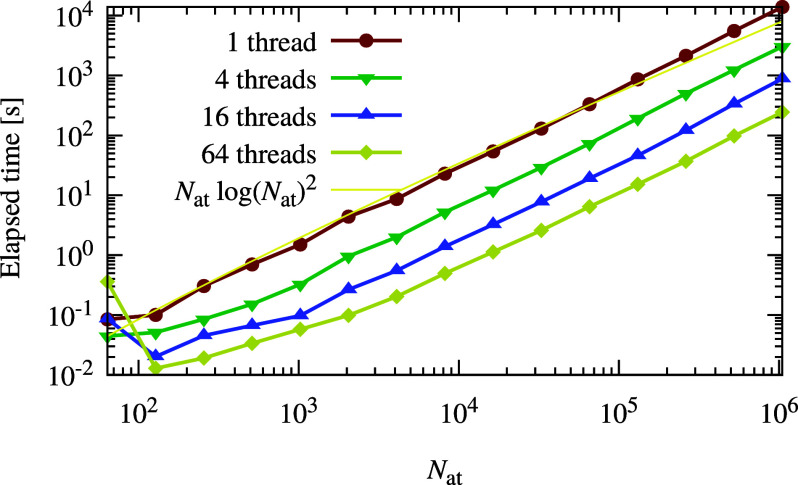
Timings of our reference implementation of iterative particle mesh charge equilibration. Shown is the time to calculate the Qeq charges for a periodic system as a function of the number of atoms in the system for different numbers of OPENMP threads. The calculations were performed on an AMD EPYC 7742 64-core processor with 1 TB of RAM. Less than 30 GB of RAM was used in total for all threads for all benchmark calculations. The fitted yellow line shows the asymptotic scaling of the computational cost of the newly developed iterative method.

Having confirmed the high performance of our new method with respect to the conventional approach, we now turn to the scaling behavior of the method. Assuming that the number of iterations needed in the conjugate gradient method does not depend on the system size, the computational costs for the iterative charge equilibration are determined by the cost of solving the electrostatic problem, which scales like  in our case. The number of conjugate gradient iterations needed to converge the charge equilibration charges slightly increases with the number of particles. Therefore, in reality the asymptotic behavior to some extent deviates from the ideal  scaling. Therefore, the charge equilibration and the computation of the derivatives have a quasi-linear scaling that is slightly higher than . Figure 2 shows the timing of our reference implementation of the iterative charge equilibration method presented in this Article. The asymptotic scaling appears to be approximately . The required calculation of the charge density and the 3-dim FFT’s can be efficiently parallelized with OpenMP, resulting in good overall parallel speed-ups. It would also be possible to use MPI-based domain decomposition to perform the charge equilibration on a parallel machine. Our OpenMP implementation is fast enough for processing systems containing tens of thousands of particles. Another interesting strategy would be to implement the iterative particle mesh charge equilibration on GPUs.

**Figure 3:**
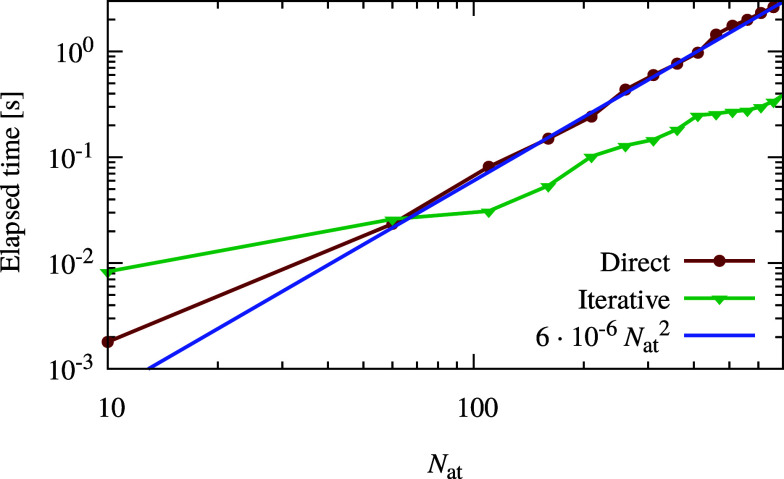
Logarithmic plot of the iterative particle mesh charge equilibration timings in comparison with the standard direct approach employing a solution of the set of linear equations. The Coulomb matrix elements were calculated using standard Ewald techniques for the direct approach, which governs the scaling for the shown small system sizes, resulting in dominantly quadratic scaling here. The calculation was done on a desktop machine with an 11th generation Intel i7 CPU (11700) and 32 GB of RAM. The fitted blue line shows the scaling of the computational cost of the direct method.

In Figure 3, the particle mesh iterative solver is compared with the standard direct approach to identify the system size at which the new approach becomes more efficient. We find that the iterative method developed in this Article is faster than the direct solution when the test system exceeds a size of about 100 atoms. The computational cost of obtaining the solution for the conventional direct approach scales cubically because of the system of equations to be solved. Since there are highly efficient solvers available for systems of linear equations, the prefactor of the cubic term is small and the quadratic term is dominant in the relatively small system size range shown in Figure 3, while the cubic scaling is anticipated to become dominant for systems between 1000 and 10000 atoms. The quadratic term in the direct approach results from calculating the matrix elements of **A**. Our newly proposed iterative method therefore outperforms the direct approach even for system sizes where the cost of solving the system of equations is negligible and the most expensive part in the direct method is calculating all of the matrix elements *A*_*ij*_. Being iterative, our approach can profit from a good input guess to reduce the number of iterations. This effect was not taken into account in our tests but will exist in many real applications. In molecular dynamics (MD), for instance, the charges from the previous MD step form a good input guess for the next MD step. This will further increase the efficiency gains of our iterative method compared to the standard direct method.

### 4.1 Validation of the Iterative Particle Mesh Charge Equilibration

The iterative particle mesh charge equilibration proposed in this Article can be used as a replacement for the current direct charge equilibration solver in the 4G-HDNNP^34^ method. Our newly developed method solves exactly the same problem and therefore predicts exactly the same charges and electrostatic energies as the clearly slower traditional direct approach from Ko et al.^34^

To accentuate the stability of our newly developed iterative charge equilibration scheme, we created a difficult artificial test system with 800 atoms consisting of 60 different elements. The atomic coordinates were chosen randomly, and the shortest distance between two atoms is 0.4 bohr. Element specific hardnesses were drawn from a uniform random distribution in the interval between 0.4 and 1.4. Electronegativities and the charges for the initial charge guess were drawn from a standard normal distribution with mean zero and variance one.

**Figure 4:**
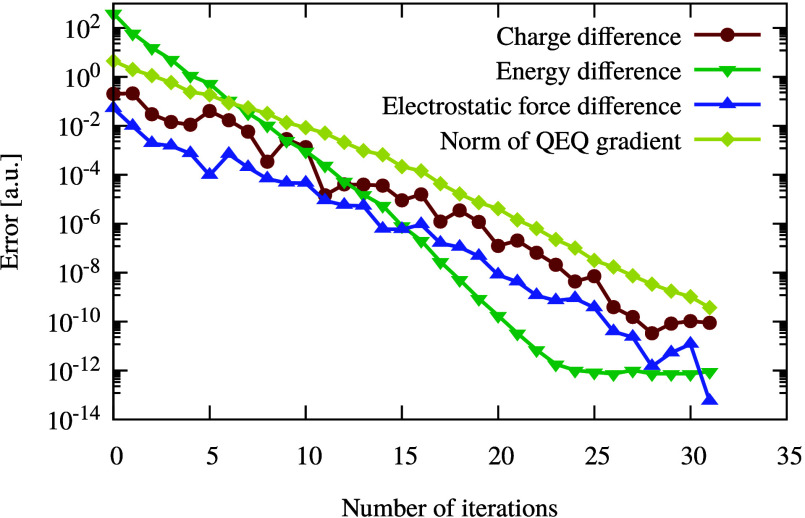
Convergence of various quantities during conjugate gradient minimization of *E*_Qeq_. The test system contains 800 atoms. To demonstrate the robustness of our method, 60 different elements, random element-specific hardnesses, and normal distributed electronegativities were used. The atomic positions were also chosen randomly, and the closest pairwise distance is 0.4 Bohr. Reference values were obtained using the direct approach. The maximum norm was used to measure the convergence of the gradient of *E*_Qeq_ with respect to that of **Q**.

As shown in Figure 4, various quantities are plotted during the minimization of *E*_Qeq_ by using the conjugate gradient method. In spite of the inherent difficulty of the test system, the CG method converges rapidly after only 31 iterations, the maximum norm of  is smaller than 10^–9^. Reference values for *E*_Qeq_, the charges, and the electrostatic approach were calculated using direct matrix inversion and Ewald techniques to calculate the Coulomb matrix. The iterative particle mesh charge equilibration was also tested and compared to realistic systems, where the convergence was even faster than in the difficult test example presented in Figure 4. This is the expected behavior, since the minimization problem from eq 3 has a unique solution and the conjugate gradient method guarantees convergence with a moderate number of steps.

This numerically confirms that our derivation of the iterative solver is correct and that one can use the newly developed iterative approach to speed up solving the charge equilibration problem in the 4G-HDNNP method.

## 5 Conclusions

In this work we have presented a quasi-linear, i.e., *N*log(*N*)^2^, scaling method for charge equilibration that allows us to speed up the evaluation of any atomistic potential that contains a charge equilibration part. The atomistic potential can be either a machine learning potential or a classical force field. The performance of our method has been investigated for the example of a fourth-generation high-dimensional neural network potential. We have shown that, due to the high efficiency of the method, it is now possible to perform simulations of systems containing thousands of atoms, which to date has been very demanding if long-range charge transfer has to be taken into account. Consequently, our method will allow to treat even complex systems with the latest generation of machine learning potentials to enable simulations of unprecedented accuracy.

## Supporting Information Available

Detailed derivations of all the steps presented in section 3 (PDF)
