# Supplementary material for: Accelerating Fourth-Generation Machine Learning Potentials Using Quasi-Linear Scaling Particle Mesh Charge Equilibration
Source: J Chem Theory Comput. 2024 Aug 16;20(16):7264–71. doi: 10.1021/acs.jctc.4c00334 (PMC11360134; doi:10.1021/acs.jctc.4c00334)
Supplement: Supplementary file 1 — ct4c00334_si_001.pdf [file ct4c00334_si_001.pdf]

Supporting Information of "Accelerating fourth-generation machine learning potentials by quasi-linear scaling particle mesh charge equilibration"

Moritz Gubler,*,† Jonas A. Finkler,† Moritz R. Schäfer,‡,¶ Jörg Behler,‡,¶ and Stefan Goedecker†

†*Department of Physics, University of Basel, Klingelbergstrasse 82, CH-4056 Basel, Switzerland*

‡*Lehrstuhl für Theoretische Chemie II, Ruhr-Universität Bochum, 44780 Bochum, Germany*

¶*Research Center Chemical Sciences and Sustainability, Research Alliance Ruhr, 44780 Bochum, Germany*

E-mail: moritz.gubler@unibas.ch

1 Derivation of charge derivatives

Using the definition of E_{elec} given in Eq. (20), $\frac{\partial E_{\text{elec}}}{\partial q_i}$ can be derived the following way:

$$\begin{aligned}\frac{\partial E_{\text{elec}}}{\partial q_i} &= \frac{1}{2}\int\int\sum_{j,k=1}^{N_{\text{at}}}\frac{\rho_j(\mathbf{r}-\mathbf{r}_j)\rho_k(\mathbf{r}'-\mathbf{r}_k)}{\|\mathbf{r}-\mathbf{r}'\|}\frac{\partial}{\partial q_i}q_j q_k d\mathbf{r}d\mathbf{r}' \\ &= \int\rho_i(\mathbf{r}-\mathbf{r}_i)\underbrace{\int\frac{\sum_{j=1}^{N_{\text{at}}}q_j\rho_j(\mathbf{r}'-\mathbf{r}_j)}{\|\mathbf{r}-\mathbf{r}'\|}d\mathbf{r}'}_{=V(\mathbf{r})}d\mathbf{r} = \int V(\mathbf{r})\rho_i(\mathbf{r}-\mathbf{r}_i)d\mathbf{r}\end{aligned} \tag{S1}$$

2 Derivation of electrostatic forces

$$\begin{aligned}\frac{\partial E(\mathbf{R}_1,\ldots,\mathbf{R}_{N_{\mathrm{at}}})}{\partial \mathbf{R}_i} &= \frac{1}{2}\int \frac{\partial}{\partial \mathbf{R}_i}\left(\rho(\mathbf{r})V(\mathbf{r})\right)d\mathbf{r}\\ &= \frac{1}{2}\int \left[V(\mathbf{r})\frac{\partial \rho(\mathbf{r})}{\partial \mathbf{R}_i} + \rho(\mathbf{r})\frac{\partial V(\mathbf{r})}{\partial \mathbf{R}_i}\right]d\mathbf{r} = \int V(\mathbf{r})\frac{\partial \rho(\mathbf{r})}{\partial \mathbf{R}_i}d\mathbf{r}. \end{aligned} \tag{S2}$$

The last part of Eq. (S2) holds because

$$\begin{aligned}\int \rho(\mathbf{r})\frac{\partial}{\partial \mathbf{R}_i}\underbrace{\int \frac{\rho(\mathbf{r}')}{\|\mathbf{r}-\mathbf{r}'\|}d\mathbf{r}'}_{=V(\mathbf{r})}d\mathbf{r} &= \int\int \frac{\rho(\mathbf{r})\frac{\partial \rho(\mathbf{r}')}{\partial \mathbf{R}_i}}{\|\mathbf{r}-\mathbf{r}'\|}d\mathbf{r}'d\mathbf{r}\\ &= \int \frac{\partial \rho(\mathbf{r}')}{\partial \mathbf{R}_i}\underbrace{\int \frac{\rho(\mathbf{r})}{\|\mathbf{r}-\mathbf{r}'\|}d\mathbf{r}}_{=V(\mathbf{r}')}d\mathbf{r}' = \int V(\mathbf{r})\frac{\partial \rho(\mathbf{r})}{\partial \mathbf{R}_i}d\mathbf{r}. \end{aligned} \tag{S3}$$

$\frac{\partial \rho}{\partial \mathbf{R}_i} = q_i \frac{\partial \rho_i}{\partial \mathbf{R}_i}$ when the definition of ρ from Eq. (2) is used. Therefore,

$$\frac{\partial E(\mathbf{r}_1,\ldots,\mathbf{r}_{N_{\mathrm{at}}})}{\partial \mathbf{r}_i} = q_i \int V(\mathbf{r})\frac{\partial \rho_i(\mathbf{r}-\mathbf{r}_i)}{\partial \mathbf{r}_i}d\mathbf{r}. \tag{S4}$$

3 Calculation of electrostatic stress

The stress tensor of the electrostatic energy is derived for periodic systems by calculating the strain derivative of the electrostatic energy in Fourier space because it requires the least number of additional Fourier transforms.

$$\begin{aligned}\frac{\partial E}{\partial \varepsilon_{\mu\nu}} &= \frac{\partial}{\partial \varepsilon_{\mu\nu}} 2\pi\Omega \sum_{\mathbf{G}\neq 0}\frac{|\tilde{\rho}(\mathbf{G})|^2}{\mathbf{G}^2}\\ &= 2\pi \underbrace{\frac{\partial \Omega}{\partial \varepsilon_{\mu\nu}}}_{\mathbf{A}}\sum_{\mathbf{G}\neq 0}\frac{|\tilde{\rho}(\mathbf{G})|^2}{\mathbf{G}^2} + 2\pi\Omega\sum_{\mathbf{G}\neq 0}\left(\frac{1}{\mathbf{G}^2}\underbrace{\frac{\partial |\tilde{\rho}(\mathbf{G})|^2}{\partial \varepsilon_{\mu\nu}}}_{\mathbf{B}} + |\tilde{\rho}(\mathbf{G})|^2 \underbrace{\frac{\partial}{\partial \varepsilon_{\mu\nu}}\frac{1}{\mathbf{G}^2}}_{\mathbf{C}}\right) \end{aligned} \tag{S5}$$

The following identities are useful for calculating the strain derivatives present in the terms **A**, **B** and **C**:

Strain derivative of a position vector
$$\frac{\partial r_\tau}{\partial \varepsilon_{\mu\nu}} = \delta_{\tau\mu} r_\nu \tag{S6}$$

Strain derivative of a Fourier space vector
$$\frac{\partial G_\tau}{\partial \varepsilon_{\mu\nu}} = -\delta_{\tau\nu} G_\mu \tag{S7}$$

Strain derivative of $\frac{1}{\mathbf{G}^2}$
$$\frac{\partial}{\partial \varepsilon_{\mu\nu}} \frac{1}{\mathbf{G}^2} = \frac{2}{\mathbf{G}^4} G_\mu G_\nu \tag{S8}$$

Strain derivative of the unit cell volume
$$\frac{\partial \Omega}{\partial \varepsilon_{\mu\nu}} = \delta_{\mu\nu} \Omega \tag{S9}$$

Strain derivative of a product of a real and a Fourier space vector
$$\frac{\partial}{\partial \varepsilon_{\mu\nu}} r_\tau G_\tau = 0 \tag{S10}$$

The Einstein summation convention is used in all of the identities. Eq. (S6) can be derived from the definition of the translation $r'_\mu = (\delta_{\mu\alpha} + \varepsilon_{\mu\alpha}) r_\alpha$. For Eq. (S7), the first Taylor expansion coefficient of the inverse transformation $((\delta_{\mu\nu} - \varepsilon_{\mu\nu}))$ can be used. The last non trivial identity is Eq. (S9). There, the useful identity $\frac{\partial \det \mathbf{M}}{\partial x} = \det \mathbf{M} \, \mathrm{Tr}\big(\mathbf{M}^{-1} \frac{\partial \mathbf{M}}{\partial x}\big)$ and the transformation law of the unit cell matrix $h'_{\mu\nu} = (\delta_{\mu\alpha} + \varepsilon_{\mu\alpha}) h_{\alpha\nu}$ is used.

The derivatives required form terms **A** and **C** are given in Eqs. (S8) and (S9) respectively. Using the product rule, term **B** can be written as $\frac{\partial |\tilde{\rho}(\mathbf{G})|^2}{\partial \varepsilon_{\mu\nu}} = \frac{\partial \tilde{\rho}(\mathbf{G}) \cdot \tilde{\rho}^*(\mathbf{G})}{\partial \varepsilon_{\mu\nu}} = \tilde{\rho}^*(\mathbf{G}) \frac{\partial \tilde{\rho}(\mathbf{G})}{\partial \varepsilon_{\mu\nu}} + \tilde{\rho}(\mathbf{G}) \frac{\partial \tilde{\rho}^*(\mathbf{G})}{\partial \varepsilon_{\mu\nu}}$ where $\frac{\partial \tilde{\rho}(\mathbf{G})}{\partial \varepsilon_{\mu\nu}}$ is the Fourier transform of $\frac{\partial \rho(x)}{\partial \varepsilon_{\mu\nu}}$. Because of Eq. (S10) $\frac{\partial e^{i\mathbf{G}\mathbf{r}}}{\partial \varepsilon_{\mu\nu}} = 0$ and therefore strain derivatives commute with the Fourier transform.

4 Charge density with periodic boundary conditions and its strain derivative

$$\rho(\mathbf{r}, \mathbf{R}_1, \ldots, \mathbf{R}_{N_{\mathrm{at}}}, \mathbf{h}) = \sum_{i,j,k=-\infty}^{\infty} \sum_{l=1}^{N_{\mathrm{at}}} q_l \rho_l \left(\underbrace{\|\mathbf{r} - \mathbf{R}_l - i\mathbf{h}_1 - j\mathbf{h}_2 - k\mathbf{h}_3\|^2}_{\mathbf{x}^2} \right) \tag{S11}$$

Only $\frac{\partial \rho_l(\mathbf{x}^2)}{\partial \varepsilon_{\mu\nu}}$ needs to be calculated to get the strain derivative or the charge density.

$$\frac{\partial \rho_l(x_\tau x_\tau)}{\partial \varepsilon_{\mu\nu}} = \frac{\partial x_\tau x_\tau}{\partial \varepsilon_{\mu\nu}} \rho_l'(\mathbf{x}^2) = 2x_\tau \frac{\partial x_\tau}{\partial \varepsilon_{\mu\nu}} \rho_l'(\mathbf{x}^2) = 2x_\tau \delta_{\tau\mu} x_\nu \rho_l'(\mathbf{x}^2) = 2x_\mu x_\nu \rho_l'(\mathbf{x}^2) \tag{S12}$$

Finally,

$$\frac{\partial \rho(\mathbf{r}, \mathbf{R}_1, \ldots, \mathbf{R}_{N_{\mathrm{at}}}, \mathbf{h})}{\partial \varepsilon_{\mu\nu}} = \sum_{i,j,k=-\infty}^{\infty} \sum_{l=1}^{N_{\mathrm{at}}} [$$
$$2q_l(\mathbf{r} - \mathbf{R}_l - i\mathbf{h}_1 - j\mathbf{h}_2 - k\mathbf{h}_3)_\mu (\mathbf{r} - \mathbf{R}_l - i\mathbf{h}_1 - j\mathbf{h}_2 - k\mathbf{h}_3)_\nu \rho_l'((\mathbf{r} - \mathbf{R}_l - i\mathbf{h}_1 - j\mathbf{h}_2 - k\mathbf{h}_3)^2)] \tag{S13}$$

or with Gaussian atomic charge densities $\rho_l(\|\mathbf{x}\|^2) = \frac{1}{\sqrt{2\pi\sigma_l^2}^3} e^{-\frac{\|\mathbf{x}\|^2}{2\sigma_l^2}}$

$$\frac{\partial \rho(\mathbf{r}, \mathbf{R}_1, \ldots, \mathbf{R}_{N_{\mathrm{at}}}, \mathbf{h})}{\partial \varepsilon_{\mu\nu}} = -\sum_{i,j,k=-\infty}^{\infty} \sum_{l=1}^{N_{\mathrm{at}}} \Bigg[$$
$$\frac{q_l}{\sqrt{2\pi\sigma_l^2}^3} \frac{1}{\sigma_l^2} (\mathbf{r} - \mathbf{R}_l - i\mathbf{h}_1 - j\mathbf{h}_2 - k\mathbf{h}_3)_\mu (\mathbf{r} - \mathbf{R}_l - i\mathbf{h}_1 - j\mathbf{h}_2 - k\mathbf{h}_3)_\nu e^{-\frac{(\mathbf{r} - \mathbf{R}_l - i\mathbf{h}_1 - j\mathbf{h}_2 - k\mathbf{h}_3)^2}{2\sigma_l^2}} \Bigg]. \tag{S14}$$

5 Charge equilibration total derivatives

Here, Eqs. (29 and (30) are simplified.

$$\sum_{i=1}^{N_{\mathrm{at}}} \lambda_i \sum_{j=1}^{N_{\mathrm{at}}} \frac{\partial A_{ij}}{\partial \mathbf{r}_k} Q_j = \sum_{i,j=1}^{N_{\mathrm{at}}} \lambda_i q_j \frac{\partial}{\partial \mathbf{r}_k} \int\int \frac{\rho_i(\mathbf{r}-\mathbf{r}_i)\rho_j(\mathbf{r}'-\mathbf{r}_j)}{\|\mathbf{r}-\mathbf{r}'\|} d\mathbf{r} d\mathbf{r}'$$

$$= \frac{\partial}{\partial \mathbf{r}_k} \int \rho^{\boldsymbol{\lambda}}(\mathbf{r}) V^{\mathbf{Q}}(\mathbf{r}) d\mathbf{r} = \int \left[V^{\mathbf{Q}}(\mathbf{r}) \frac{\partial \rho^{\boldsymbol{\lambda}}(\mathbf{r})}{\partial \mathbf{r}_k} + V^{\boldsymbol{\lambda}}(\mathbf{r}) \frac{\partial \rho^{\mathbf{Q}}(\mathbf{r})}{\partial \mathbf{r}_k} \right] d\mathbf{r} \quad \text{(S15)}$$

$$\sum_i \lambda_i \sum_j \frac{\partial A_{ij}}{\partial \varepsilon_{\mu\nu}} Q_j = \frac{\partial}{\partial \varepsilon_{\mu\nu}} \int \rho^{\boldsymbol{\lambda}}(\mathbf{r}) V^{\mathbf{Q}}(\mathbf{r}) d\mathbf{r} = \frac{\partial}{\partial \varepsilon_{\mu\nu}} 2\pi\Omega \sum_{\mathbf{G}\neq 0} \rho^{\boldsymbol{\lambda}*}(\mathbf{G}) \tilde{V}^{\mathbf{Q}}(\mathbf{G})$$

$$= 2\pi\Omega \sum_{\mathbf{G}\neq 0} \left[\delta_{\mu\nu} \rho^{\boldsymbol{\lambda}*}(\mathbf{G}) \tilde{V}^{\mathbf{Q}}(\mathbf{G}) + \frac{\partial \tilde{\rho}^{\boldsymbol{\lambda}*}(\mathbf{G})}{\partial \varepsilon_{\mu\nu}} \tilde{V}^{\mathbf{Q}}(\mathbf{G}) + \tilde{\rho}^{\boldsymbol{\lambda}*}(\mathbf{G}) \frac{\partial \tilde{V}^{\mathbf{Q}}(\mathbf{G})}{\partial \varepsilon_{\mu\nu}} \right]$$

$$= 2\pi\Omega \sum_{\mathbf{G}\neq 0} \left[\delta_{\mu\nu} \rho^{\boldsymbol{\lambda}*}(\mathbf{G}) \tilde{V}^{\mathbf{Q}}(\mathbf{G}) + \frac{\partial \tilde{\rho}^{\boldsymbol{\lambda}*}(\mathbf{G})}{\partial \varepsilon_{\mu\nu}} \tilde{V}^{\mathbf{Q}}(\mathbf{G}) + \frac{\tilde{\rho}^{\boldsymbol{\lambda}*}(\mathbf{G})}{\mathbf{G}^2} \left(\frac{\partial \tilde{\rho}^{\mathbf{Q}}(\mathbf{G})}{\partial \varepsilon_{\mu\nu}} + \frac{2}{\mathbf{G}^2} G_\mu G_\nu \tilde{\rho}^{\mathbf{Q}}(\mathbf{G}) \right) \right]$$

$$= 2\pi\Omega \sum_{\mathbf{G}\neq 0} \frac{1}{\mathbf{G}^2} \left[\delta_{\mu\nu} \rho^{\boldsymbol{\lambda}*}(\mathbf{G}) \tilde{\rho}^{\mathbf{Q}}(\mathbf{G}) + \frac{\partial \tilde{\rho}^{\boldsymbol{\lambda}*}(\mathbf{G})}{\partial \varepsilon_{\mu\nu}} \tilde{\rho}^{\mathbf{Q}}(\mathbf{G}) \right.$$

$$\left. + \tilde{\rho}^{\boldsymbol{\lambda}*}(\mathbf{G}) \left(\frac{\partial \tilde{\rho}^{\mathbf{Q}}(\mathbf{G})}{\partial \varepsilon_{\mu\nu}} + \frac{2}{\mathbf{G}^2} G_\mu G_\nu \tilde{\rho}^{\mathbf{Q}}(\mathbf{G}) \right) \right] \quad \text{(S16)}$$
